# Transcriptome Analysis of Pseudomonas aeruginosa Biofilm Infection in an *Ex Vivo* Pig Model of the Cystic Fibrosis Lung

**DOI:** 10.1128/aem.01789-21

**Published:** 2022-02-08

**Authors:** Niamh E. Harrington, Jenny L. Littler, Freya Harrison

**Affiliations:** a School of Life Sciences, Gibbet Hill Campus, The University of Warwick, Coventry, United Kingdom; Novo Nordisk Foundation Center for Biosustainability

**Keywords:** biofilm, chronic infection, cystic fibrosis, *ex vivo* model, *Pseudomonas aeruginosa*, RNA sequencing, transcriptome, quorum sensing, antimicrobial resistance, RNAseq, antibiotic resistance, biofilms

## Abstract

Pseudomonas aeruginosa is the predominant cause of chronic biofilm infections that form in the lungs of people with cystic fibrosis (CF). These infections are highly resistant to antibiotics and persist for years in the respiratory tract. One of the main research challenges is that current laboratory models do not accurately replicate key aspects of a P. aeruginosa biofilm infection, highlighted by previous RNA-sequencing studies. We compared the P. aeruginosa PA14 transcriptome in an *ex vivo* pig lung (EVPL) model of CF and a well-studied synthetic cystic fibrosis sputum medium (SCFM). P. aeruginosa was grown in the EVPL model for 1, 2, and 7 days, and *in vitro* in SCFM for 1 and 2 days. The RNA was extracted and sequenced at each time point. Our findings demonstrate that expression of antimicrobial resistance genes was cued by growth in the EVPL model, highlighting the importance of growth environment in determining accurate resistance profiles. The EVPL model created two distinct growth environments: tissue-associated biofilm and the SCFM surrounding tissue, each cuing a transcriptome distinct from that seen in SCFM *in vitro*. The expression of quorum sensing associated genes in the EVPL tissue-associated biofilm at 48 h relative to *in vitro* SCFM was similar to CF sputum versus *in vitro* conditions. Hence, the EVPL model can replicate key aspects of *in vivo* biofilm infection that are missing from other current models. It provides a more accurate P. aeruginosa growth environment for determining antimicrobial resistance that quickly drives P. aeruginosa into a chronic-like infection phenotype.

**IMPORTANCE** Pseudomonas aeruginosa lung infections that affect people with cystic fibrosis are resistant to most available antimicrobial treatments. The lack of a laboratory model that captures all key aspects of these infections hinders not only research progression but also clinical diagnostics. We used transcriptome analysis to demonstrate how a model using pig lungs can more accurately replicate key characteristics of P. aeruginosa lung infection, including mechanisms of antibiotic resistance and infection establishment. Therefore, this model may be used in the future to further understand infection dynamics to develop novel treatments and more accurate treatment plans. This could improve clinical outcomes as well as quality of life for individuals affected by these infections.

## INTRODUCTION

The Gram-negative, facultative anaerobe and opportunistic pathogen Pseudomonas aeruginosa is the most common cause of chronic biofilm infection in the lungs of people with the genetic condition cystic fibrosis (CF), associated with reduced life quality and increased mortality (1). The pathogen’s ability to persist for years in the respiratory tract of people with CF is associated with its adaptive mechanisms including the switch to a biofilm lifestyle, conferring protection to the host immune response and high antibiotic tolerance (2). Biofilm formation creates a spatial organization that results in complex cell-to-cell interactions and increased infection population heterogeneity (3). Although these infections are widely regarded as the best described and most studied biofilm infection in medicine, there are no completely effective eradication strategies (4). There are a number of limitations with the current laboratory models, and these have impacted research progression (5, 6). The lack of an *in vivo*-like growth platform for current susceptibility testing methods to prescribe drugs often results in poor clinical outcomes (7).

Existing infection models of the CF lung include mouse, ferret, and pig models, as well as biofilms formed on beads or epithelial cultures and laboratory sputum media designed to mimic human CF sputum. The variety of different models, each capturing different features of CF lung infection, has led to RNA sequencing (RNA-seq) studies that aim to identify the transcriptomic aspects of P. aeruginosa chronic biofilm infection not captured by current work (8, 9). This approach has highlighted the importance of growth environment in determining P. aeruginosa traits involved in virulence and persistence and antimicrobial resistance (AMR), key considerations for treating CF lung infections (8–10). A quantitative computational framework using transcriptomic data for the evaluation of human infection model accuracy has also been developed (5). This work found that an *in vitro* CF epithelial cell model and a revised version of a specific synthetic cystic fibrosis sputum medium (SCFM2) both resulted in P. aeruginosa gene expression more comparable to the P. aeruginosa transcriptome from human CF sputum than other models tested. The key aspects of *in vivo* metabolism captured included fatty acid and phospholipid metabolism in the epithelial cell model, and nucleoside and nucleotide metabolism in SCFM2. However, there are aspects of infection that are not reproduced by these two models. Alginate production and quorum sensing (QS)-associated genes were among the genes that caused the biggest distinction between *in vitro* and *in vivo* transcriptomes (both overexpressed *in vitro*) (5). Both alginate and QS are directly linked to P. aeruginosa persistence and virulence (11, 12); thus, there is a crucial gap in our ability to reproduce CF-like phenotypes in the laboratory.

We have developed an *ex vivo* pig lung (EVPL) model of the CF lung environment that replicates key phenotypic aspects of P. aeruginosa chronic biofilm infection (13–16). We aimed to assess the P. aeruginosa transcriptome as biofilm infection establishes in the EVPL model, to determine the extent to which the model accurately replicates P. aeruginosa gene expression during human CF infection. We show that the EVPL model creates two environments that are distinct from SCFM *in vitro* growth and that it cues patterns of P. aeruginosa gene expression that are more comparable to those seen in human infection. When the transcriptome of P. aeruginosa grown as a biofilm on the EVPL tissue is compared with that of P. aeruginosa in SCFM *in vitro*, the changes in expression of key metabolic pathways, quorum sensing-controlled virulence determinants, and genes linked to antibiotic resistance are similar to those observed when the P. aeruginosa transcriptome *in vivo* in CF infection is compared with the *in vitro* transcriptome.

## RESULTS

The EVPL model comprises two environments: the bronchiolar lung tissue surface (lung) and the SCFM that surrounds the tissue (surrounding SCFM) to mimic the luminal mucus in the human CF lung (see Fig. S1). We aimed to determine the changes in the P. aeruginosa PA14 transcriptome cued by growth as either tissue-associated biofilm or, in the surrounding SCFM of the EVPL model, compared with SCFM *in vitro.* PA14 was grown in the EVPL model and in SCFM alone for 7 days, and comparisons were made at two time points (24 h and 48 h; we could not recover sufficient mRNA from 7-day populations in SCFM *in vitro*). We used these comparisons to address the overall question: does biofilm growth on pig bronchiolar tissue better replicate the P. aeruginosa transcriptome observed in human infection than growth in SCFM *in vitro*?

### EVPL tissue surface maintains viable P. aeruginosa populations for longer than SCFM *in vitro*.

P. aeruginosa PA14 was grown on 3 replica tissue sections from each of two independent lungs for 24 h, 48 h, or 7 days. RNA was extracted from the lung-associated biofilm and the surrounding SCFM for each sample. RNA was also extracted at the same time points from PA14 grown in SCFM *in vitro*. At 7 days, RNA concentrations sufficient for sequencing could not be consistently obtained from P. aeruginosa grown *in vitro* (Table S1 in the supplemental material). However, sufficient P. aeruginosa RNA was obtained from lung tissue-associated biofilm at this time point. We confirmed that PA14 was still viable on the lung tissue at 7 days postinfection (PI) (Fig. S2) and did not appear to be visibly “stressed” as seen for *in vitro* cultures (rounding of cells; Fig. S3). Hence, RNA from lung tissue-associated biofilms was sequenced from 24 h, 48 h, and 7 days PI, but RNA from *in vitro* cultures and the SCFM surrounding lung tissue was sequenced from 24 h and 48 h only.

CFU were measured from representative repeats for each condition used for RNA-seq to check for consistent growth between environments (Fig. S4). The presence of PA14 was confirmed by distinct morphology on the Luria-Bertani (LB) agar plates used to determine CFU. Briefly, recovered CFU was comparable between *in vitro* SCFM and the lung tissue at 24 h and 48 h PI, and this density was maintained at 7 days PI on the lung tissue. The CFU ml^−1^ recovered from the surrounding SCFM was approximately one log_10_ higher than that recovered from either the tissue-associated biofilm or *in vitro* cultures at both 24 h and 48 h PI. However, across all environments and time points, the PA14 CFU was consistent with the high densities and variability of CFU that are recovered from people with CF (Fig. S4): chronic P. aeruginosa lung infection has an expected bacterial load of 10^8^ to 10^10^ ml^−1^ (17).

### P. aeruginosa transcriptome analysis reveals distinct niches in the EVPL model compared with *in vitro* SCFM, and distinct changes in transcriptome over time.

Following initial data preparation, all reads from EVPL environments (lung and surrounding SCFM) were aligned to the pig genome to exclude any contaminating porcine RNA. There were ≤ 1% of reads aligned to the pig genome across all samples (median 0.09%; see Table S2). Subsequently, remaining EVPL model reads and *in vitro* SCFM reads were successfully aligned to the P. aeruginosa PA14 genome (median 98.9%; Table S2), proving the bacterial RNA extracted was predominantly P. aeruginosa PA14. This confirmed that there were few metabolically active endogenous microbes present in the EVPL tissue.

**FIG 1:**
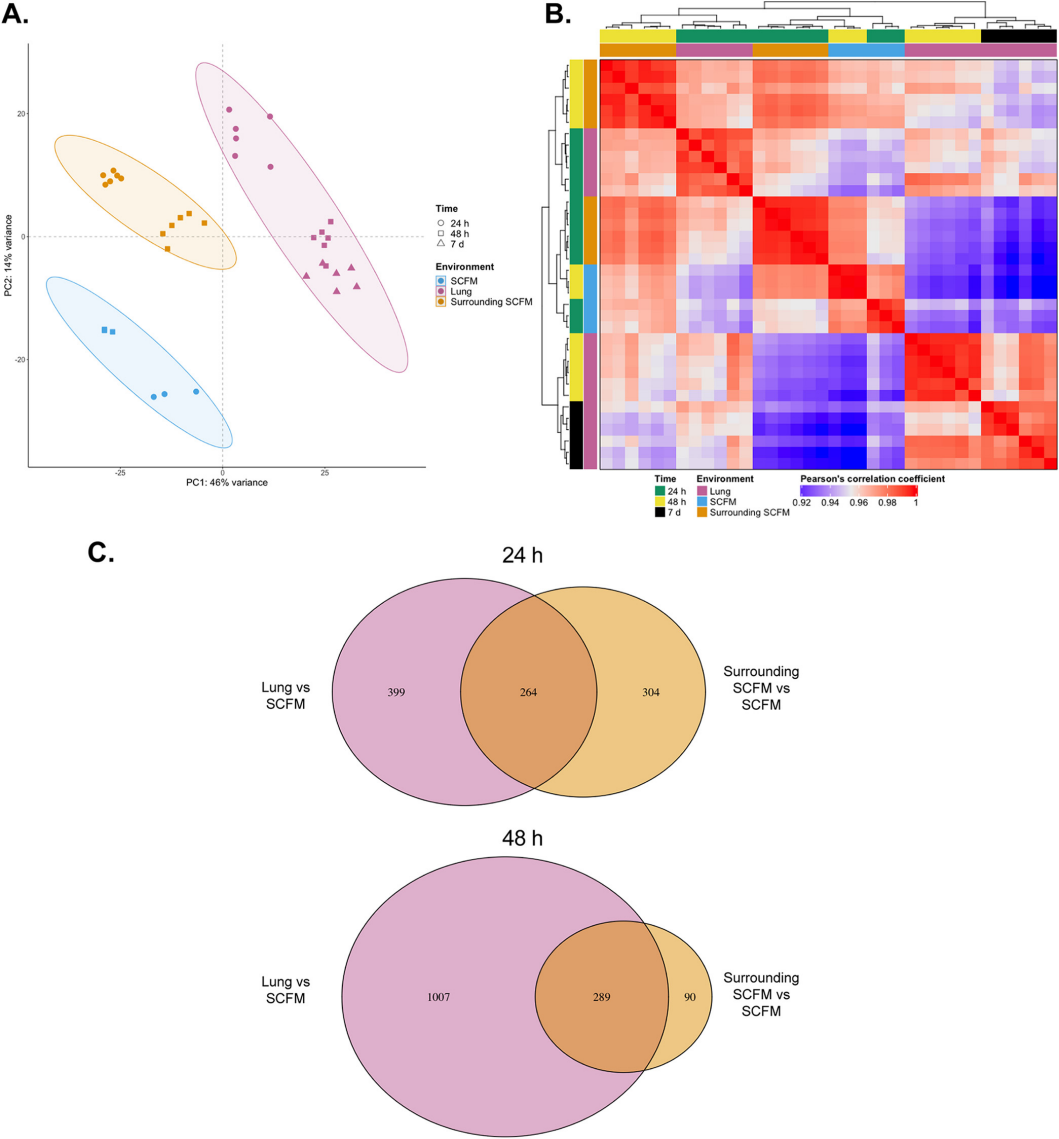
Initial investigation of the Pseudomonas aeruginosa PA14 transcriptome across 3 environments: *in vitro* synthetic cystic fibrosis sputum media (SCFM) and the two niches in the *ex vivo* pig lung model: lung tissue surface (lung) and the SCFM surrounding the lung tissue (surrounding SCFM), based on the whole genome (*n* = 5829 genes). (A) Principal-component analysis (PCA) considering all genes. Each environment is shown by a different color and each time point shown by different shaped points (see key). The 95% confidence ellipses are shown. Individual data points represent each RNA sample; three tissue pieces from each of two independent pig lungs were used for the EVPL model environments at each time point, and three replica *in vitro* SCFM cultures were sequenced per time point. (B) Heatmap showing hierarchical clustering analysis and the Pearson’s correlation coefficient value between each sample (all *r *> 0.9). The P. aeruginosa PA14 growth environment and infection time for each sample is shown by different combinations of colors (see key). (C) Venn diagrams of the number of significant differentially expressed P. aeruginosa PA14 genes (DEGs) from each contrast, using threshold values of *P < *0.05 and log_2_ fold change ≥ |1.5|. The shared DEGs are genes that are either underexpressed or overexpressed in both the lung and surrounding SCFM versus *in vitro* SCFM. Genes that were significant DEGs in both contrasts at each time point, but in opposite directions, are not considered to be shared between both contrasts. The full list of significant DEGs is provided in the data supplemental material.

Initial principal-component analysis (PCA), considering 5829 genes, demonstrated that the different environments in the EVPL model resulted in distinct P. aeruginosa PA14 expression profiles (Fig. 1A). This suggests that the environmental cues the bacteria encounter may differ between lung tissue-associated biofilm and the airway mucus, resulting in two distinct infection populations. There was also a difference in how expression changed over time when PA14 was grown in the EVPL model or in SCFM *in vitro*. As shown in Fig. 1A, the shift in the first two principal components from 24 h to 48 h (and 7 days in the lung-associated biofilm) followed opposite directions for *ex vivo* growth compared with SCFM *in vitro*. This highlights that as well as distinct overall differences in P. aeruginosa gene expression between the three environments, there was also a difference in how the expression profile changed over time as the populations established. Subsequent Pearson’s correlation coefficient analysis (*r *> 0.9 for all comparisons) and hierarchical clustering (Fig. 1B) showed that the transcriptome of PA14 grown on lung tissue at the two later time points (48 h and 7 days) was distinct from PA14 in the surrounding SCFM at 24 h, and growth in SCFM *in vitro* at both time points (Fig. 1B).

Differential expression analysis was then performed to identify significant differentially expressed genes (DEGs) for each contrast (*P < *0.05, log_2_ fold change ≥ |1.5|). Comparison of the two *ex vivo* environments (lung and surrounding SCFM) and *in vitro* SCFM at 24 h and 48 h PI found a number of significant DEGs shared between lung-associated biofilm and surrounding SCFM. There were also many significant DEGs specific to each EVPL model environment (Fig. 1C). Similar numbers of genes were significantly differentially expressed in both the lung-associated P. aeruginosa biofilm population and surrounding SCFM, compared with *in vitro*, at 24 h PI (663 and 568 genes, respectively). Of these, 264 significant DEGs were common to both populations. At 48 h PI there was a comparable number of DEGs shared between the lung and surrounding SCFM, versus *in vitro* SCFM, to 24 h (Fig. 1C). However, the overall number of significant P. aeruginosa DEGs in the lung tissue biofilm compared with *in vitro* SCFM had increased (1,296 genes) (Fig. 1C). This trend was not observed in the surrounding SCFM (decrease to 379 overall DEGs; 90 unique). These findings reveal clear differences in PA14 gene expression between the EVPL model and *in vitro* SCFM and indicate that this difference grows as the biofilm establishes over time on the lung tissue surface. The difference is maintained from 48 h to 7 days PI, with these time points appearing to be more comparable in the number of significant DEGs than 24 h versus 7 d (Fig. S5). In contrast, the significant difference between *in vitro* growth and the surrounding SCFM population appears to reduce over time. This indicates that as a P. aeruginosa biofilm infection establishes in the EVPL model, the difference between the PA14 transcriptome in the surrounding SCFM and *in vitro* SCFM becomes less distinct, while the difference between lung-associated biofilm and *in vitro* becomes more distinct.

### Gene expression differences in EVPL tissue-associated P. aeruginosa biofilms versus *in vitro* SCFM growth demonstrate functional importance.

Differential expression analysis found significant differences in the P. aeruginosa transcriptome depending on whether it was growing as a biofilm associated with pig lung tissue, in the SCFM surrounding the lung tissue, or *in vitro* in SCFM. We therefore determined the functional importance of these differences at the two time points that we were able to study across all 3 environments: 24 h and 48 h PI. KEGG pathway enrichment analysis identified multiple pathways that were significantly enriched (*P < *0.05) in the contrasts between both EVPL environments and *in vitro* SCFM growth at 24 h PI, including quorum sensing (both *P < *0.05; see Fig. S6 for full results). There were also significant KEGG pathways unique to each comparison: cationic antimicrobial peptide resistance and biofilm formation were significantly enriched in the lung-associated biofilm compared with *in vitro* SCFM (both *P < *0.05). The results indicated that the significant DEGs between comparisons have functional context that may cause different infection phenotypes.

**FIG 2:**
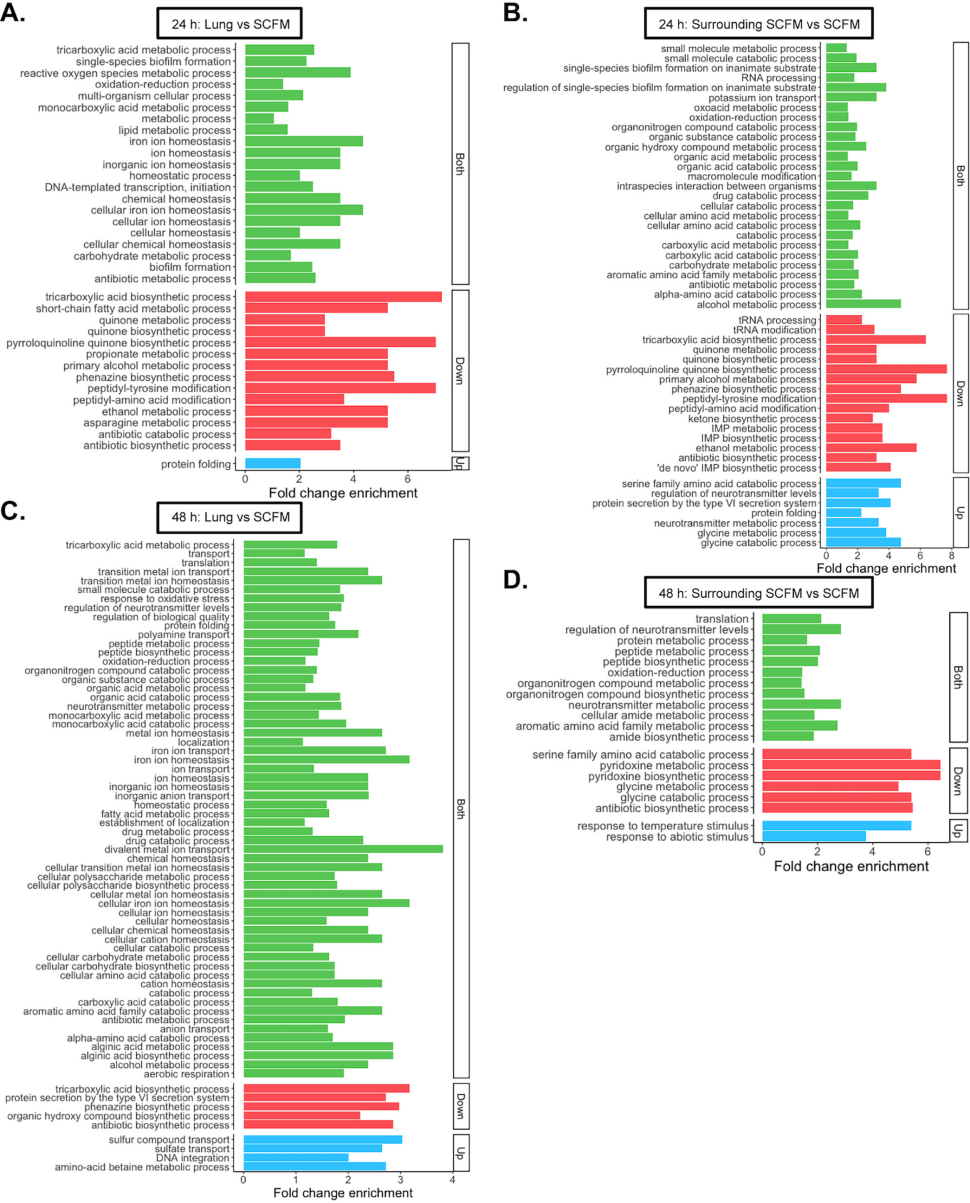
Significantly enriched Pseudomonas aeruginosa PA14 biological processes gene ontology (GO) terms (*P < *0.05, log_2_ fold change ≥ |1.5|) from contrasts between growth from each of the two *ex vivo* pig lung model locations: the lung tissue (lung) and the synthetic cystic fibrosis sputum media (SCFM) surrounding the lung tissue (surrounding SCFM), and *in vitro* SCFM. The analysis was performed on samples from 24 h postinfection (A, B) and 48 h postinfection (C, D). Each graph shows the significantly enriched biological processes GO terms for the particular contrast on the *y* axis and fold change enrichment on the *x* axis. The fold change enrichment is the fold difference in expression of significant differentially expressed genes (DEGs) in the analysis associated with that GO term than expected by random chance. Green bars show terms where the associated significantly DEGs were either overexpressed or underexpressed, showing that the process was affected irrespective of the direction of expression. The red bars show terms where all associated significant DEGs in the contrast were underexpressed, and blue bars show terms where all associated significant DEGs were overexpressed.

Gene ontology (GO) term analysis was then performed to provide more detailed functional information (significantly enriched: *P < *0.05). Fig. 2 shows the PA14 biological process GO terms that were significantly enriched in the lung-associated biofilm and surrounding SCFM compared with *in vitro* SCFM, at 24 h and 48 h PI. The number of significantly enriched GO terms increases over time in the lung versus *in vitro* SCFM; however, it decreases in the surrounding SCFM comparison (Fig. 2). This is consistent with our previous conclusion that there was a reduction in number of significant DEGs in surrounding SCFM versus *in vitro* SCFM from 24 h to 48 h PI, and an increase in significant DEGs in the lung-associated biofilm (Fig. 1C). Additionally, the GO term “response to abiotic stimulus” was significantly enriched in the surrounding SCFM compared with *in vitro* SCFM 48 h PI (*P = *0.04; Fig. 2D). All associated genes were upregulated in the surrounding SCFM. It is possible this is a result of interaction at the interface between the SCFM and the SCFM-agarose pad placed in the tissue culture plates to support the bronchiolar tissue section. The same 24-well plates were used for *in vitro* growth, so the plate surface is a consistent condition and unlikely to cause expression differences. This GO term was not found to be significantly enriched in the lung-associated PA14 biofilm at either time point (Fig. 2A and C).

The GO term “phenazine biosynthetic process” was found to be significantly enriched in the P. aeruginosa PA14 lung biofilm versus *in vitro* SCFM growth at both time points (Fig. 2A and C). The KEGG pathway “phenazine biosynthesis” was also found to be significantly enriched for these comparisons (Fig. S6). All genes for the biosynthesis of the exotoxin pyocyanin were downregulated in the lung-associated biofilm at 48 h compared with *in vitro* SCFM (Fig. S6). This suggests that phenazine biosynthesis, particularly pyocyanin production, may be an important differentiation between *in vitro* and pig lung grown P. aeruginosa biofilms.

### P. aeruginosa quorum-sensing-associated pathways are downregulated in the EVPL model compared with *in vitro* SCFM growth, as observed in CF sputum samples.

Quorum sensing (QS) pathways in P. aeruginosa have long been considered to play a role in the establishment of biofilms (18). Three key P. aeruginosa QS systems are associated with controlling biofilm formation in human infection: the LasI/R system, RhlI/R, and the Pseudomonas quinolone signal (PQS) (12, 19, 20). These acyl-homoserine lactone (AHL) associated systems regulate the expression of numerous genes associated with virulence and chronic biofilm infection (12). We found that the QS KEGG pathway was significantly enriched in the SCFM surrounding P. aeruginosa infected EVPL tissue versus *in vitro* SCFM growth at 24 h and 48 h PI, and in the lung biofilm versus *in vitro* at 24 h PI (Fig. S6). The majority of the significant DEGs in this pathway were underexpressed in the lung environments compared with *in vitro* (Table 1). These results, alongside QS being an extensively researched area with the potential as a new therapeutic target in CF (21, 22), led to more detailed exploration of the expression of QS-associated genes in the EVPL model.

**TABLE 1:**
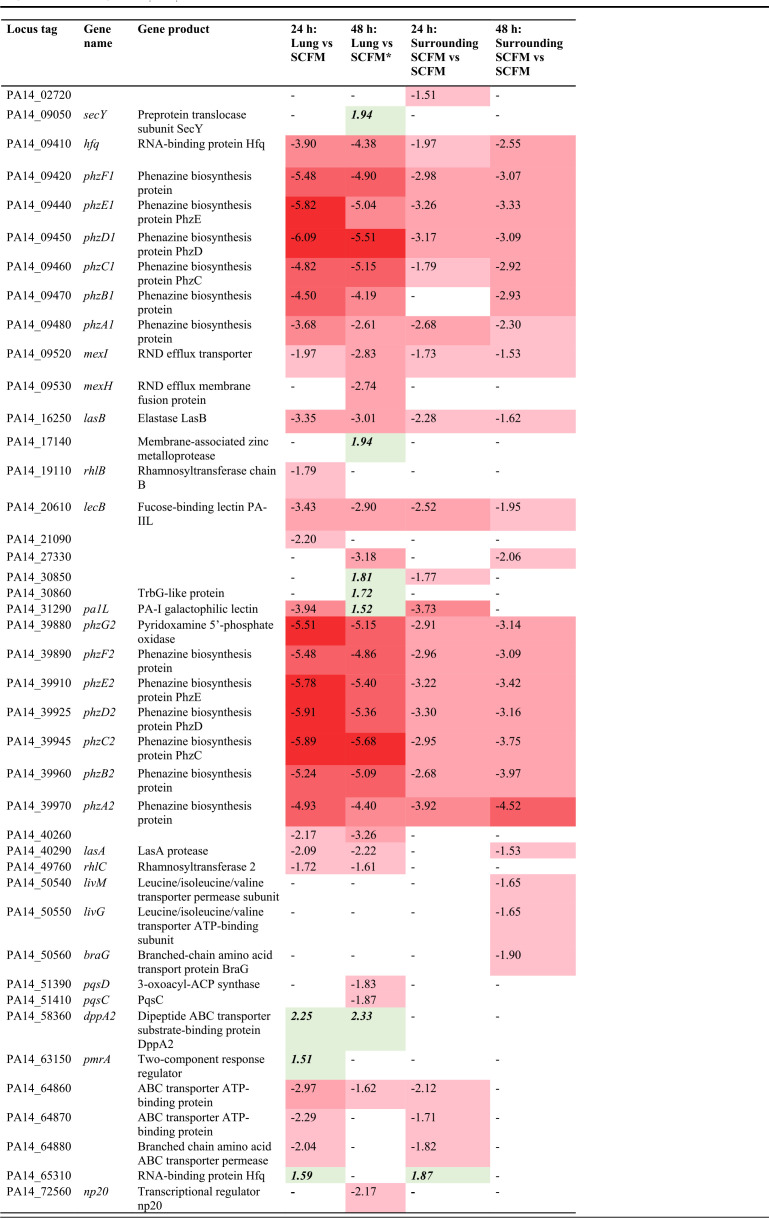
All genes associated with the Pseudomonas aeruginosa PA14 KEGG pathway quorum sensing as listed on *pseudomonas*.com (51) that were found to be significantly differentially expressed (*P < *0.05, log_2_ fold change ≥ |1.5|) in at least one contrast^*a*^

a PA14 expression in the two locations of the *ex vivo* pig lung model: the lung tissue-associated biofilm (lung) and the synthetic cystic fibrosis sputum media (SCFM) surrounding the lung tissue (surrounding SCFM) were compared with *in vitro* SCFM growth. Samples were compared at 24 h and 48 h postinfection. The log_2_ fold change (LFC) is shown where significant, and contrasts denoted with “-” for a gene show it was not significantly differentially expressed. The red fill shows genes that are underexpressed (also standard font) in the EVPL environments versus *in vitro* SCFM; the darker the color, the greater the LFC. The green fill shows genes that are overexpressed in the EVPL environments (bold and italicized font). The * denotes the only contrast where the quorum sensing KEGG pathway was not significantly enriched.

Further investigation of the QS-associated significant DEGs demonstrated that most of these genes are in the phenazine biosynthesis operons, as shown in Table 1 (for full list see Table S3). This finding further supports these pathways as key to the difference between *ex vivo* and *in vitro* grown P. aeruginosa transcriptomes. These genes had a greater log_2_ fold change reduction on the lung-associated biofilm compared with *in vitro* SCFM at 24 h and 48 h PI, than surrounding SCFM versus *in vitro*. The expression of *pqsC* and *pqsD*, both important in the production of PQS (23), was also found to be underexpressed in the lung compared with *in vitro* SCFM 48 h PI (Table 1). This suggests that this third QS system is also being downregulated over time in the lung-associated biofilm in comparison with *in vitro* SCFM.

**FIG 3:**
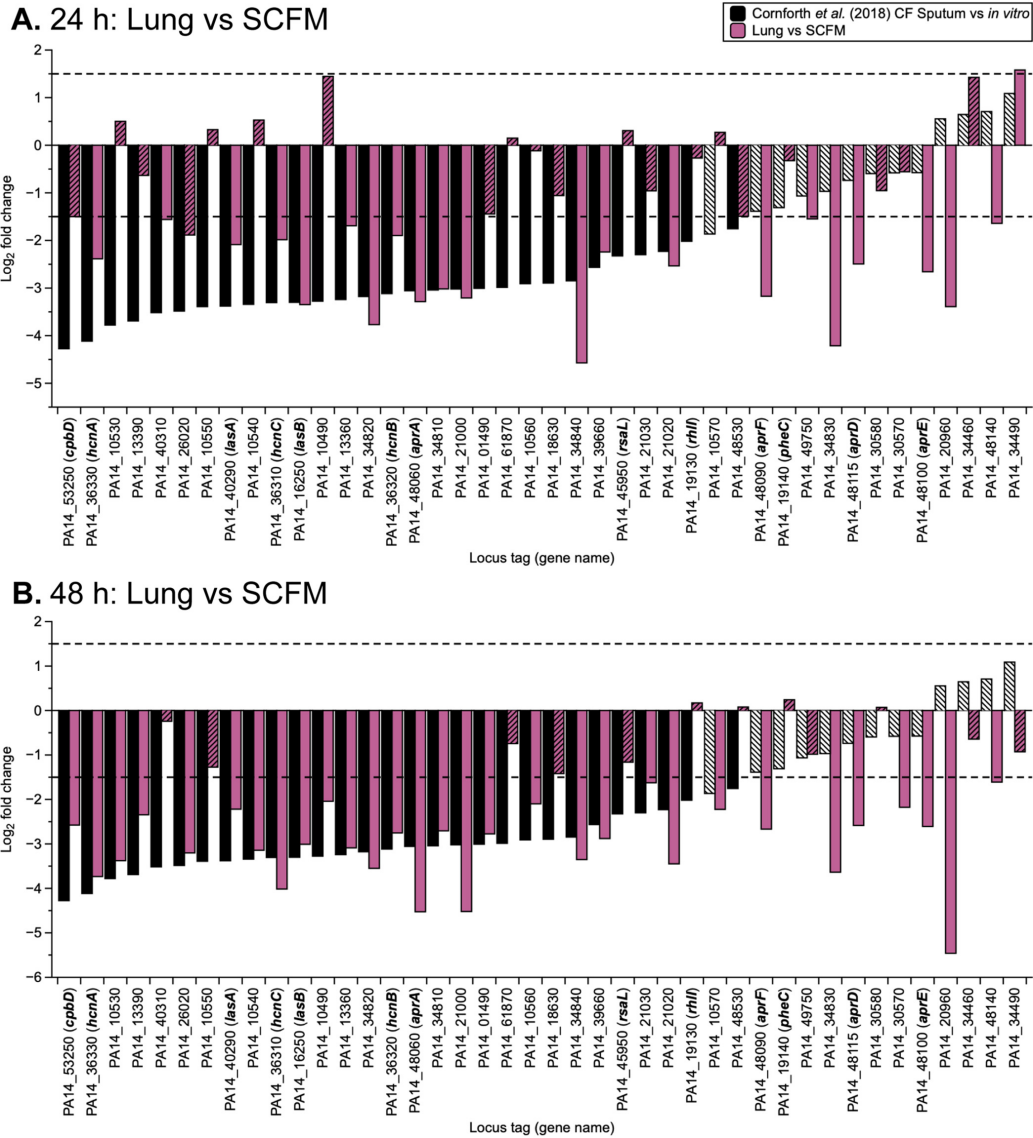
The log_2_ fold change (LFC) in expression of 42 Pseudomonas aeruginosa quorum sensing genes, controlled by the las regulon, conserved in human infection. The expression of P. aeruginosa PA14 grown on the lung tissue of the *ex vivo* pig lung tissue (Lung) versus *in vitro* synthetic cystic fibrosis media (SCFM) at 24 h (A) and 48 h (B) postinfection are shown by the purple bars. Each graph also includes expression of the gene set by P. aeruginosa from human cystic fibrosis sputum versus *in vitro* conditions taken from Cornforth et al. (8), shown by the black bars. The locus tags shown are for P. aeruginosa PA14 with gene names in bold where appropriate. Bars with the striped fill are not significantly differentially expressed for that contrast (*P < *0.05, LFC ≥ |1.5|). The dashed lines represent the threshold LFC value for differential expression.

The study of P. aeruginosa transcriptomes by Cornforth et al. (8) found that expression of a set of conserved genes under las-regulation differs between *in vitro* growth and human infection, with lower levels of expression observed *in vivo*. This set of 42 P. aeruginosa genes regulated by the las QS system were found to be conserved among CF lung isolates (24). Thus, these genes were specifically investigated in our RNA-seq results. The expression of the equivalent gene set in P. aeruginosa PA14 when grown on *ex vivo* pig lung tissue compared with *in vitro* SCFM showed similar significant differential expression to CF sputum versus *in vitro* as found by Cornforth et al. (8) (Fig. 3). At 24 h PI similar changes in expression of these genes were found in the EVPL tissue-associated biofilm compared with SCFM *in vitro* (Fig. 3A), as seen in human CF sputum (*lasA*: −2.09 and −3.38 log_2_ fold change, respectively, and *lasB*: –3.35 and –3.30). At 48 h PI, the expression profile of this gene set becomes even more comparable to the relative expression in CF sputum (Fig. 3B). These genes were also studied in surrounding SCFM growth versus *in vitro* SCFM at both time points, and although the pattern was less obvious, there were some similarities with expression in CF sputum versus *in vitro* (Fig. S7). These findings support the conclusion that both environments of the EVPL model more closely capture a key aspect of human CF P. aeruginosa infection than *in vitro* growth, but the lung tissue associated biofilm at 48 h appears to be most representative.

**FIG 4:**
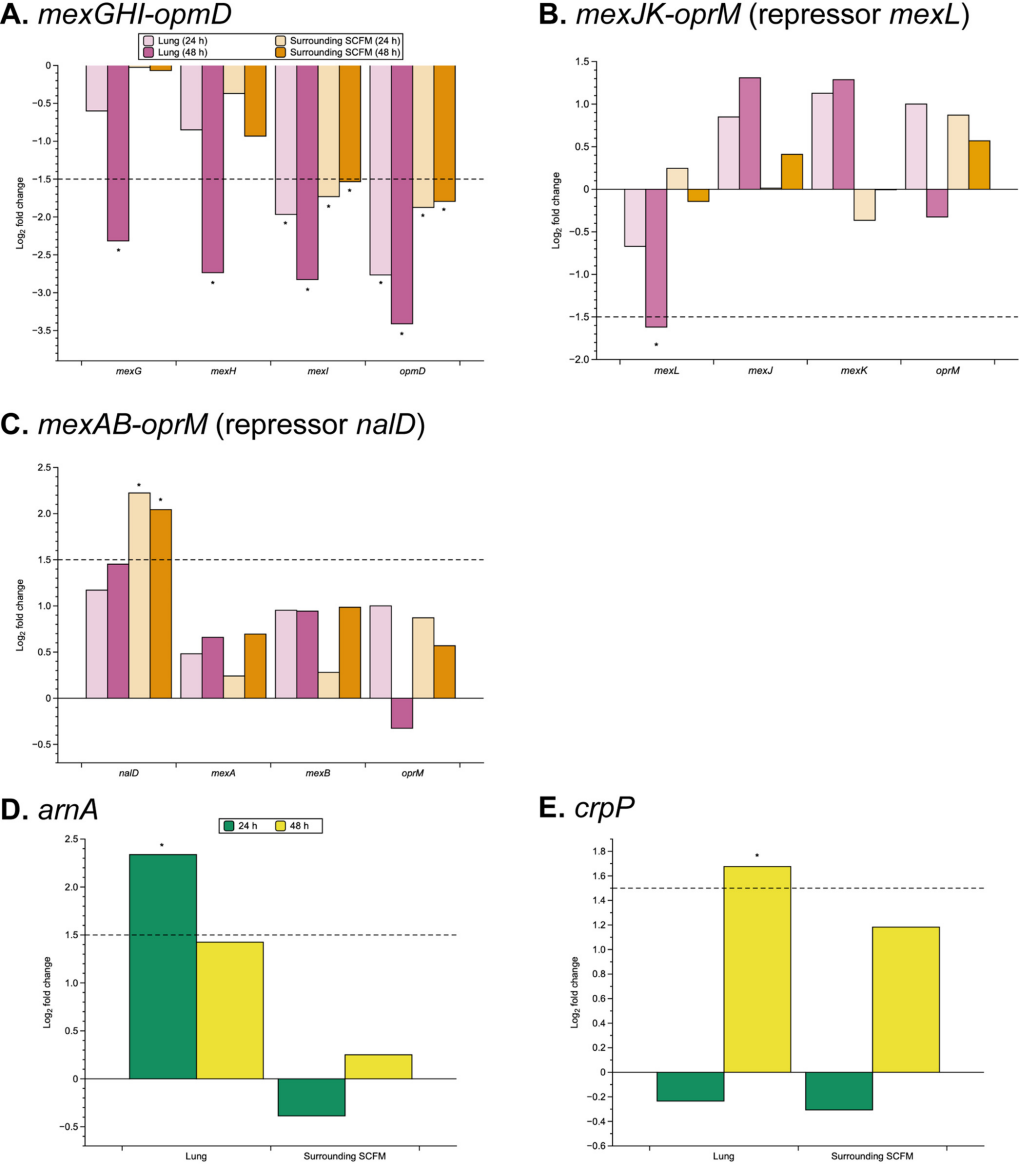
The log_2_ fold change (LFC) of genes of interest associated with antibiotic resistance predicted by the Comprehensive Antibiotic Resistance Database (CARD) (26). Comparisons of gene expression were performed for the two environments of the *ex vivo* pig lung model: the lung tissue and surrounding synthetic cystic fibrosis media (SCFM) versus *in vitro* SCFM at two time points (24 h and 48 h postinfection). The dashed lines represent the threshold LFC value for a gene to be considered significantly differentially expressed (LFC ≥ |1.5|), and comparisons where this was statistically significant (*P < *0.05) are denoted with a *. Each bar color represents a different comparison and time point (see keys). (A–C) The LFC values for each comparison of efflux pumps and any repressors where significant expression differences were found in at least one comparison. (D–E) The LFC values for each comparison of individual genes where significant expression differences were found in at least one comparison.

Alongside our RNA-seq findings, we performed phenotypic assays to measure the concentration of extracellular 3-oxo-dodecanoyl homoserine lactone (3-oxo-C12-HSL) in each environment at 24 h and 48 h PI. Production of 3-oxo-C12-HSL is encoded by the gene *LasI*; it binds to the LasR transcriptional regulator to regulate expression of numerous genes (20). We found a significantly lower concentration of extracellular 3-oxo-C12-HSL in both *ex vivo* conditions compared with *in vitro* SCFM at 48 h PI (Fig. S8), consistent with the downregulation of the *las*-controlled gene set. However, neither *lasI* nor *lasR* were found to be significantly differentially expressed for any comparisons (Table S3). A previous study by Aendekerk et al. (25) that investigated the MexGHI-OpmD efflux pump found that mutations in this pump resulted in P. aeruginosa being unable to produce 3-oxo-C12-HSL. RT-PCR showed that this lack of 3-oxo-C12-HSL production did not correlate with inhibition of *lasI* or *rhlI* transcription; thus, MexGHI-OmpD exerts its effects on 3-oxo-C12-HSL levels post-transcriptionally. This mechanism is consistent with our results, as we found that all genes encoding MexGHI-OpmD were significantly underexpressed in the lung-associated biofilm compared with *in vitro* SCFM growth at 48 h PI (Fig. 4A). Genes associated with self-degradation of acyl homoserine lactone (AHL) signals were not found to be significantly overexpressed in any of our comparisons (Table S4). The expression of the MexAB-OprM pump components, which transport 3-oxo-C12-HSL out to the cell, was also not significantly different in the lung-associated biofilm (Fig. 4C).

### Genes associated with antibiotic resistance are differentially expressed in EVPL environments compared with *in vitro* SCFM.

Following investigation of quorum sensing gene expression and the implication on efflux pump expression, we aimed to determine whether growth in the EVPL model—with no prior exposure to antimicrobials—resulted in differing expression of antimicrobial resistance (AMR) associated genes. We have previously shown that high levels of antibiotic tolerance are evident in the EVPL model (16) and clinical infection typically demonstrates much higher resistance to antimicrobial treatment than is predicted *in vitro*. We looked at the expression of 52 P. aeruginosa PA14 AMR genes, predicted using the Comprehensive Antibiotic Resistance Database (CARD) (26), in each contrast. There was significant differential expression of 22 AMR associated genes in the two EVPL model environments: lung tissue and surrounding SCFM, compared with *in vitro* SCFM growth of PA14 (see Table S5).

Three efflux pumps were of particular interest in at least one of the comparisons (Fig. 4A to C). All genes encoding the efflux pump MexG/HI-OpmD were found to be significantly underexpressed in the lung-associated biofilm compared with *in vitro* SCFM growth at 48 h PI (Fig. 4A). MexHI-OpmD is associated with resistance to fluoroquinolones, whereas MexGHI-OpmD is associated with the post-transcriptional regulation of 3-oxo-C12-HSL production as described above, and exerts effects on antibiotic sensitivity via its effects on QS (27). The transcriptional repressor of the MexJK-OprM efflux pump (*mexL*), linked to triclosan resistance (28), was also found to be significantly underexpressed in the lung-associated biofilm compared with *in vitro* SCFM (Fig. 4B). Conversely, a transcriptional repressor of the MexAB-OprM efflux pump (*nalD*) was significantly overexpressed in the surrounding SCFM at 24 h and 48 h compared with *in vitro* SCFM. Previously, mutations in repressors of this efflux pump have been linked to carbapenem resistance and overexpression of MexAB-OprM (29). However, this pump has also been proven to no longer be required upon the formation of a mature biofilm (30), which may suggest why a transcriptional repressor is being overexpressed in the surrounding SCFM as a P. aeruginosa biofilm is establishing in the EVPL model (15).

Two individual genes of interest were found to be significantly differentially expressed in at least one comparison (Fig. 4D and E). Both genes (*arnA* and *crpP*) are associated with the *arn* locus, linked with resistance to cationic antimicrobial peptides (CAMPs) (31). They were both found to be significantly differentially expressed in the lung tissue associated biofilm compared with *in vitro* SCFM: *arnA* overexpressed at 24 h PI and *crpP* at 48 h. This was consistent with MIC values for colistin and polymyxin B (CAMPs) of P. aeruginosa PA14 cells from the lung tissue associated biofilm found to be increased compared with SCFM and the standard cation-adjusted Mueller-Hinton broth (CAMHB) at both time points (Table S6). These findings indicated that growth of PA14 biofilm on EVPL tissue causes changes in expression of AMR pathways, compared with *in vitro* SCFM, that have a phenotypic effect.

## DISCUSSION

P. aeruginosa infection of the CF lung is one of the most well-studied biofilm infection contexts, yet it remains highly resistant to the most aggressive antimicrobial treatments available and eventually results in loss of lung function and accelerated death (1, 3). Research to improve prevention measures and treatment of chronic CF infection is hindered by the lack of accurate laboratory growth conditions and models to truly mimic biofilm formation as observed in human infection. It has been shown that growth environment plays an integral role in the P. aeruginosa transcriptome; P. aeruginosa gene expression in CF sputum samples has a distinct transcriptional profile compared with current laboratory models including mouse models and an enhanced, updated version of SCFM (SCFM2) (8–10). We compared gene expression in our EVPL model, considering the tissue-associated biofilm and bacteria in the surrounding SCFM as separate populations, with gene expression in SCFM *in vitro*. We focused on key aspects of infection that may not be well captured by current laboratory models: quorum sensing and antibiotic resistance. Characteristics of P. aeruginosa chronic infection were observed in the model as early as 1 day postinfection. This was much faster than the accumulation of mutations, observed over years, important for P. aeruginosa adaptation to form an established chronic infection in the CF lung (32). We propose that our previously described EVPL model can be used to mimic P. aeruginosa biofilm infection as seen in CF not only in structure (15) but also in key aspects of the transcriptome.

We compared gene expression across the whole genome in both environments of the EVPL model, and in SCFM *in vitro*, across multiple time points. Our analyses revealed that the lung tissue and surrounding SCFM of the EVPL model create two distinct niches from each other and from *in vitro* cultures. To date, development of laboratory model systems has predominantly focused on recapitulating CF sputum through production of artificial sputum media (33). However, sputum media such as SCFM and SCFM2, discussed above, do not attempt to recapitulate the tissue-associated biofilm infection that also occurs in the CF airway. Our results show a clear difference in P. aeruginosa gene expression between a synthetic sputum and lung tissue of the EVPL model. This highlights the tissue associated biofilm as a distinct niche that is arguably just as important to further understand infection dynamics as the “sputum-like” population. A clear distinction in gene expression changes in the population over time in *ex vivo* conditions versus *in vitro* was also found. Thus, as well as growth environment being important for expression differences at each point of comparison, it also affected the dynamics of infection progression.

The greatest difference between *in vitro* and *ex vivo* conditions was in the lung tissue-associated biofilm (versus *in vitro* SCFM) at 48 h PI. In particular, genes in phenazine biosynthetic pathways were found to differ in expression. Phenazines are redox-active pigments with antimicrobial activity that are known to cause changes in gene expression and antibiotic susceptibility (34). The phenazine pyocyanin is an important P. aeruginosa virulence factor, and all genes in its biosynthetic pathway were found to be underexpressed in the lung tissue environment at 48 h compared with *in vitro*. Virulence factor production is typically downregulated during the chronic stages of infection; in fact, overproduction of pyocyanin during CF infection is considered an unusual phenotype (35). Thus, these findings indicate that the EVPL model is causing a P. aeruginosa phenotype reminiscent of CF chronic infection that is not seen *in vitro*. This may allow for the study of established biofilms, which are typically difficult to treat stage of infection.

Phenazines are also signaling molecules, and regulation of the associated operons involves all three P. aeruginosa QS systems: *las*, *rhl*, and *pqs* (36). QS has been shown to be a key aspect of infection where current laboratory studies cue overexpression upon comparison to human infection (8). We found that the shift in expression of conserved *las* controlled genes in the lung-associated biofilm versus *in vitro* SCFM at 48 h PI is almost identical to the shift in expression of these genes in CF sputum versus *in vitro* (8). These results suggest that the EVPL model could more accurately mimic the QS expression profile seen in human infection than other current laboratory models. While QS is important in the initial stages of infection, an accumulation of mutations in QS pathways are observed over time in CF, suggesting that it is not as active in the later stages of infection (11). Interestingly, *lasI/R* and *rhlI/R* were not found to be significantly differentially expressed in any of our comparisons; the differences were in downstream las- and rhl-regulated genes. A reduced concentration of extracellular 3-oxo-C12-HSL produced by P. aeruginosa was also measured in the *ex vivo* environments compared with *in vitro* SCFM growth at 48 h PI. P. aeruginosa mutants for the efflux pump-associated genes *mexI* and *opmD* have previously been shown to not produce 3-oxo-C12-HSL due to an intracellular accumulation of a toxic PQS precursor due to loss of pump activity (25). We found that both genes were downregulated in the lung and surrounding SCFM versus *in vitro* SCFM growth, indicating that the reduction in production of the QS molecule is likely caused by reduced expression of the efflux pump. Interestingly, genes that encode amidases able to degrade AHL signals (37) (PA14 homologues of *pvdQ*, *quiP*, and *hacB*) were not found to be differentially expressed between the EVPL model and SCFM at either time point. Thus, our results indicate that the reduction in extracellular 3-oxo-C12-HSL is not due to quorum quenching activity and is likely a reduction in production driven by the downregulation of MexGHI-OpmD. Hence, the differences in QS expression between P. aeruginosa grown in the EVPL model and *in vitro* SCFM may be part of a wider network that also has implications for antibiotic resistance. Future proteomics work would determine whether this was the true cause of the difference.

Increased antibiotic resistance is a characteristic of P. aeruginosa infection of the CF lung that poses significant clinical concern. We found a number of efflux pump genes and resistance-associated operons to be significantly differentially expressed in the EVPL model. The downregulation of the MexGHI-OpmD efflux pump in the tissue-associated biofilm at 48 h, as discussed above, may be linked to increased antibiotic resistance as well as QS effects. Mutations in *mexI* and *opmD* have previously been shown to increase P. aeruginosa resistance to aminoglycosides, β-lactams, and quinolones (25). A transcriptional regulator of the MexAB-OprM pump (*nalD*) was found to be overexpressed in the surrounding SCFM compared with *in vitro* SCFM, indicating a reduction in this pump also. However, no significant difference in any of the efflux pump genes or the repressor were found in the lung-associated biofilm, as was the case for the other efflux pumps found. The spatial arrangement of MexAB-OprM has been previously proven to be heterogenous within infection populations, with a higher proportion of the pump found in cells that formed a dense biofilm (30). This may explain why our results indicate there are higher levels of the efflux pump in the lung-associated population than surrounding SCFM. This further distinction between the surrounding SCFM and lung tissue suggests these two spatially linked environments promote different patterns of gene expression that may result in varying antibiotic resistance phenotypes. The model provides a heterogenous infection population that may express different mechanisms of resistance that cannot be captured by *in vitro* SCFM alone. This is an important consideration for determining treatment approaches and novel treatments for P. aeruginosa infection in the CF lung.

The overexpression of *arnA* in the lung-associated biofilm at 24 h PI and *crpP* at 48 h is also of clinical interest, as both genes are linked to CAMP resistance. The gene *arnA* is part of the *arn* locus that functions via lipid A modifications increasing resistance (31). Upregulation of this gene at 24 h PI but not 48 h may be providing resistance prior to biofilm formation, after which point the biofilm matrix acts as a protection mechanism against antibiotic treatment. In contrast, *crpP* is upregulated at 48 h PI. CrpP has been shown to confer resistance to ciprofloxacin via phosphorylation (38). The upregulation of this gene in the tissue-associated biofilm despite no antibiotic treatment suggests this pathway is initiated by growth environment, not antibiotic exposure. As well as providing further insight into resistant infection, these findings highlight the importance of using a clinically relevant infection model, such as the EVPL, to better understand the resistance profile of bacteria *in vivo*. Conversely, we must consider that people with CF receive numerous antibiotic treatment courses, so the resistance profiles of clinical infection will be impacted by this as well as the host environment.

We have specifically focused on the expression of genes associated with QS and AMR to demonstrate how the tissue associated biofilm captures characteristics of *in vivo* infection not observed in other models. Although SCFM has been shown to be a good laboratory model for CF sputum, these two aspects of infection are pathways where SCFM does not accurately recapitulate P. aeruginosa expression *in vivo* (8). While no model is perfect, our results show that the EVPL model can more closely capture these aspects of infection than SCFM alone. To address future research hypotheses using the EVPL model, this RNA sequencing data can be used to ensure specific pathways are being regulated as expected and show the model is suitable to ask specific research questions.

In conclusion, we have demonstrated that the EVPL model creates two environments of interest, tissue-associated biofilm and the surrounding SCFM, that are distinct from *in vitro* SCFM. Gene expression in the tissue-associated biofilm at 48 h appears to be more representative of an established infection of the CF lung. We have focused on expression at a whole population level; however, future expression studies at the single-cell level may reveal further insight into the infection dynamics and population heterogeneity to improve development of novel, effective treatments. Future studies could also focus on transcriptomics of clinical P. aeruginosa isolates in the EVPL model, as their response to the environment may differ from that of PA14 (9). These studies have the capacity to be conducted over longer timescales than studies conducted on P. aeruginosa *in vitro* in SCFM; by 7 days PI, we could not recover mRNA from cells grown *in vitro* and bacteria appeared stressed, but EVPL-grown cells were viable and yielded good quality mRNA. Although endogenous species did not survive when EVPL tissue was infected with PA14, interaction with the lung microbiome as well as other pathogens is a key factor in P. aeruginosa infection in CF, which could be explored further in the future. Overall, the EVPL model is a useful platform to dissect key aspects of P. aeruginosa pathophysiology in CF within days of infection, which has not been possible in current *in vitro* models.

## MATERIALS AND METHODS

### Bacterial strain.

The wild type Pseudomonas aeruginosa PA14 strain used in this study was from the University of Washington (39, 40). It was grown on Luria-Bertani (LB) agar (Melford Laboratories) for 24 h at 37°C prior to all infections and to determine CFU. The pSB1075 Escherichia coli biosensor, which carries a fusion of *lasRI*’::*luxCDABE* (41), was used to measure 3-oxo-C12-HSL in culture supernatants.

### Synthetic cystic fibrosis sputum medium.

The synthetic cystic fibrosis sputum medium (SCFM) was prepared based on a previously published recipe (33), with the glucose removed. Preliminary work found that with the addition of porcine lung tissue, glucose facilitated endogenous bacterial growth, and the presence or absence of glucose did not affect P. aeruginosa growth (14).

### *Ex vivo* pig lung dissection and infection.

All pig lungs used were supplied by a commercial butcher (Steve Quigley & Sons, Cubbington, United Kingdom) and dissected on the day of arrival from the abattoir. *Ex vivo* pig lung (EVPL) tissue was dissected to extract the bronchioles as previously described (14, 15, 42). Following UV light sterilization, the square bronchiolar tissue pieces were placed into each well of a 24-well plate(s) with a 400 μL, UV-sterilized, 0.8% (wt/vol) agarose pad.

To infect each tissue piece, a 29 G sterile hypodermic needle (Becton, Dickinson Medical) was used to touch the surface of a colony of the infection strain from an overnight LB plate and then transferred to the tissue by lightly piercing the surface. To “mock inoculate” the uninfected negative control pieces, a sterile needle was used. To fully replicate the CF lung environment, 500 μL SCFM was added to each tissue-containing well and the plate covered with a Breathe-Easier membrane (Diversified Biotech) sterilized with UV. Plates were incubated stationary for the required length of time at 37°C.

### Bacterial recovery from the EVPL model and bacterial count determination.

There were two environments in the EVPL model: the bronchiolar tissue (tissue) and the SCFM surrounding each tissue piece (surrounding SCFM). To recover bacteria from the tissue, each tissue piece was removed from the 24-well plate following incubation and transiently washed in 500 μL phosphate-buffered saline (PBS). Tissue sections were then placed in sterile homogenization tubes (Fisherbrand); each had 18 2.38 mm metal beads (Fisherbrand) and 1 ml PBS added. To recover the biofilm-associated population, the tissue-containing tubes were bead beat using a FastPrep-24 5G (MP Biomedicals) for 40 s at 4 m s^−1^. The surrounding SCFM was directly transferred to individual sterile 2-ml DNA lo-bind tubes from the 24-well plate (Eppendorf).

To determine bacterial load, an aliquot was taken from each tissue homogenate and surrounding SCFM sample and serially diluted in PBS. Dilutions were plated on LB agar and incubated at 37°C for 24 h. Tissue and surrounding SCFM taken from the same sample were recorded as the same repeat number for comparison. Colony counts were performed, and the CFU per lung and per ml for the surrounding SCFM were calculated.

### Microbial cell viability from EVPL tissue.

The BacTiter-Glo microbial cell viability assay (Promega) was used on EVPL tissue-associated samples across a 7-day infection period. Cell viability was measured by the amount of ATP (nM) produced. Following lung dissection, infection, incubation, and recovery, the lung homogenate was equilibrated to room temperature. Once room temperature was reached, 100 μL of each sample was added to each well of a 96-well black plate. The assay was performed as per kit instructions using a Tecan Spark 10M multimode plate reader, and ATP (nM) was determined using the ATP (Jena Bioscience) standard curve produced.

### P. aeruginosa *in vitro* SCFM growth.

P. aeruginosa colonies from an overnight LB agar plate were suspended in an appropriate volume of SCFM to an OD_600nm_ of 0.05. Aliquots of 1 mL were added to each well of a 24-well plate and covered with a UV-sterilized Breathe-Easier membrane. Plates were incubated stationary at 37°C for the required length of time.

### Production of 3-oxo-C12-HSL.

The lung homogenate, surrounding SCFM, and *in vitro* SCFM cultures were filter sterilized using a 0.2-μm pore syringe filter (Fisherbrand) into sterile 2-mL Eppendorf tubes. The sterile supernatants were then stored at −20°C prior to performing the assay.

The E. coli pSB1075 bioreporter for 3-oxo-C12-HSL was grown in 10 ml LB broth (+10 μg mL^−1^ tetracycline) overnight at 37°C, with 170 rpm shaking. The overnight culture was then diluted 1:100 in 15 mL LB broth and incubated at 37°C, with 170 rpm shaking for 3.5 h. The culture was centrifuged at 13,000 rpm for 2 min and the supernatant discarded. The pellet was then resuspended in 15 mL PBS. The centrifugation and resuspension were repeated twice more, and the final pellet resuspended in 15 mL LB. The OD_600nm_ was adjusted to 0.1 using LB broth. Sterile sample supernatants were defrosted on ice, diluted 1 in 10 in PBS, and mixed 1:1 with the final E. coli culture to a volume of 200 μL, in a 96-well black plate. A standard curve was also performed in the same plate, using 3-oxo-C12-HSL (Sigma-Aldrich) concentrations from 1 nM to 0.000001 nM in 10-fold increments. The plate was then incubated at 37°C for 7.5 h in a Tecan Sark 10M multimode plate reader, and the OD_600nm_ and relative light units (RLU) were read every 15 min. The RLU/OD was calculated, and the final concentrations of 3-oxo-C12-HSL were determined at the inflection point, using the standard curve values.

### MIC assay.

Lung homogenate was diluted 1 in 100 in SCFM to be used as the bacterial inoculum to perform MIC assays (MICs). The inoculum for MICs in SCFM and cation-adjusted CAMHB were prepared as a MacFarland standard. Briefly, P. aeruginosa PA14 was grown on an LB agar plate overnight, and colonies were suspended in the relevant media to an OD_600nm_ of 0.08–0.1 and then diluted 1 in 100.

MICs were performed following the broth microdilution method as described by Wiegand et al. (43). The antibiotics, colistin (Acros Organics) and polymyxin B (Sigma-Aldrich), were serially diluted 1 in 2, from 128–0.0125 μg mL^−1^ to a final volume of 100 μL, in tissue culture-treated 96-well plates (Corning) using the relevant media. Subsequently, 100 μL of bacterial inoculum was added to each antibiotic well as well as positive control wells with no antibiotic added. Negative control wells were prepared with only water. Plates were then incubated at 37°C for 18 h. The lowest antibiotic concentration where no growth was visible was recorded as the MIC value.

### P. aeruginosa RNA extraction.

P. aeruginosa RNA was extracted from three infection environments: EVPL tissue, EVPL surrounding SCFM, and *in vitro* SCFM. Three tissue pieces from each of two independent pig lungs were used for the EVPL environments and three replica *in vitro* SCFM cultures, each per time point. The cultures of interest were transferred to individual sterile 2-ml DNA lo-bind tubes: 1 mL *in vitro* culture per tube, 500 μL surrounding SCFM per tube, and 1 mL lung homogenate per tube. Subsequently, 0.5 volume sterile killing buffer (20 mM Tris-HCl pH 7.5, 5 mM MgCl_2_, 20 mM NaN_3_) was added to each tube and then centrifuged at 13,000 rpm for 1 min. The samples were snap-frozen and stored at −80°C for at least 1 h.

Samples were defrosted on ice and the supernatant gently removed, ensuring the pellet remained in-tact. Each pellet was resuspended in 600 μL sterile LETS buffer (0.1 M LiCl, 0.01 M Na_2_EDTA, 0.01 M Tris-HCl pH 7.5, 0.2% SDS) and transferred to 2-ml lysing matrix B tubes (MP Biomedicals). Tubes were bead beat using a FastPrep-24 5G for three cycles: 6 m s^−1^ for 40 s then 5 min incubation on ice. The samples were further incubated in the lysing matrix B tubes until they reached room temperature, centrifuged for 10 min at 13,000 rpm, and 600 μL 125:24:1 Phenol Chloroform Isoamyl alcohol (PCI) pH 4.5 (Invitrogen) was added. Each tube was vortexed for 5 min at ∼14,000 rpm then centrifuged at 15,000 rpm for 5 min at 4°C. The top layer of solution was transferred to sterile 2-ml RNase-free tubes (Sarstedt Ltd) and 1 volume of 125:24:1 PCI pH 4.5 added. The previous vortex, centrifuge, and top layer transfer steps were repeated, and 1 volume 24:1 Chloroform Isoamyl alcohol (Sigma-Aldrich) was added. All samples were then centrifuged at 15,000 rpm for 5 min and the top layer transferred to a new 2-ml RNase-free tube. Finally, 0.1 volume of 3M NaCH_3_COO pH 5.2 and 1 volume of isopropanol were added and mixed by inverting each tube. Samples were stored at −20°C overnight.

### RNA precipitation and DNA removal.

RNA samples were defrosted on ice and centrifuged at 15,000 rpm for 15 min. The supernatant was removed, and each pellet resuspended in 1 volume 70% (vol/vol) ethanol. Resuspended samples were centrifuged at 15,000 rpm for 15 min at 4°C. Supernatant was removed and pellets left to dry for ∼15 min by a flame, then resuspended in 50 μl RNase-free water. Each sample was incubated on ice for 3 h then for a further 30 min at room temperature. The precipitated RNA concentration was determined using the Qubit RNA BR (broad range) assay kit. All RNA was snap-frozen and stored at −80°C.

Frozen RNA was defrosted and any samples above 200 μg mL^−1^ were diluted in RNase-free water to ≤ 200 μg mL^−1^. Each sample was transferred into PCR tubes, to a maximum of 50 μL per tube; samples with a higher volume were divided into multiple tubes. A 9:1 ratio of DNase I buffer (10×) (Invitrogen) to RNA sample respectively was added to each tube. Subsequently, 2 μL DNase I (Invitrogen) was added to each reaction and the tubes incubated at 37°C for 30 min. A further 2 μL DNase I was added, and the incubation repeated. An equal volume ethanol to sample was added, thoroughly mixed, then transferred into Zymo-Spin ICC columns (Direct-zol RNA MiniPrep Plus kit, Zymo Research). Samples separated due to high concentration were combined in one spin column. Spin columns were centrifuged at 13,000 rpm for 30 s and the flow-through discarded. Each RNA sample was cleaned up using the Direct-zol RNA MiniPrep Plus kit. For the final elution, tubes were incubated at 55°C for 5 min to increase RNA yield. A 16S PCR and gel electrophoresis were used to confirm complete DNA removal, and for any samples with detectable DNA, the protocol was repeated. Final RNA concentration was confirmed using the Qubit RNA BR assay kit. Samples were snap-frozen and stored at −80°C.

### Quality check of RNA and sequencing.

Total extracted RNA quality was confirmed for sequencing using the Agilent 2100 Bioanalyzer system using the RNA 6000 Pico Kit (Agilent). Bacterial and mammalian rRNA depletion, and Illumina library preparation for strand-specific RNA-sequencing, was performed by Genewiz, then samples were sequenced on an Illumina NovaSeq 150 bp paired-end run.

### Bioinformatic analyses.

RNA-seq reads were initially quality checked using FastQC v0.11.8 (44), which was subsequently used after each step of initial data preparation. Reads were trimmed using Trimmomatic v0.38 (45), with a minimum read length threshold of 25 bp (8). Any residual bacterial rRNA transcripts, and eukaryotic where appropriate, were filtered out using SortmeRNA v2.1b (46). All reads from EVPL-grown samples (surrounding SCFM and lung) were mapped to the pig genome (Sus scrofa: NCBI, GCF_000003025.6) using HISAT2 v2.1.0 (47), and the aligned reads were removed from the trimmed reads using Seqtk v1.3-r106 (48). The remaining EVPL sample reads and *in vitro* SCFM sample reads were aligned to the P. aeruginosa UCBPP-PA14 genome (NCBI, assembly GCF_000014625.1) using the BWA v0.7.17-r1188 aligner with the MEM algorithm (49). Reads mapped to coding sequences were counted using the “featureCounts” function from the R package Rsubread v2.0.1 (50) against the P. aeruginosa UCBPP-PA14 annotation sourced from *pseudomonas*.com (51). XCount data were normalized using the rlog transformation function from the R package DESeq2 v1.26.0 (52), then used for principal-component analysis (PCA) based on all genes in the analysis (5,829) using the “plotPCA” function within DESeq2. The 95% confidence ellipses were determined and added to the PCA plot using ggpubr v0.4.0 (53). A hierarchical clustering heatmap was produced to show pairwise correlation values based on Pearson’s correlation coefficient for all sample comparisons using the “HeatmapAnnotation” function from the ComplexHeatmap v2.2.0 R package (54).

Differential gene expression analysis between samples from different growth environments and time points was performed using DESeq2; genes were considered significantly differentially expressed with an adjusted *P* value < 0.05 (Benjamini-Hochberg procedure to control the false discovery rate) and log_2_ fold change (FC) ≥ |1.5|. KEGG pathway enrichment analysis of the differentially expressed genes (DEGs) was carried out with the “enrichKEGG” function from the R package clusterProfiler v3.14.3 (55) with the P. aeruginosa UCBPP-PA14 organism KEGG code (“pau”). KEGG pathways were considered significantly enriched using an adjusted *P* value < 0.05 (Benjamini-Hochberg). Gene ontology (GO) term enrichment analysis was also performed using topGO v2.38.1 (56). Reported significantly enriched GO terms were based on Fisher’s exact test *P* value < 0.05. Antimicrobial resistance genes investigated were sourced from *pseudomonas*.com (51), based on predictions by the Comprehensive Antibiotic Resistance Database (CARD) (26).

### Data Availability.

A full list of significant DEGs from each contrast represented in Fig. 1C is provided as a data supplement, and all raw sequence data are available at the European Nucleotide Archive (accession number: PRJEB48552).
